# Metabolic Engineering and Comparative Performance Studies of *Synechocystis* sp. PCC 6803 Strains for Effective Utilization of Xylose

**DOI:** 10.3389/fmicb.2015.01484

**Published:** 2015-12-24

**Authors:** Saurabh Ranade, Yan Zhang, Mecit Kaplan, Waqar Majeed, Qingfang He

**Affiliations:** ^1^Department of Biology, University of Arkansas at Little RockLittle Rock, AR, USA; ^2^Biotechnology Research Center, Shandong Academy of Agricultural SciencesJinan, China; ^3^Center for Integrative Nanotechnology Sciences, University of Arkansas at Little RockLittle Rock, AR, USA

**Keywords:** cyanobacteria, metabolic engineering, mixotrophy, LAHG, *Synechocystis*, xylose transporter

## Abstract

Wood sugars such as xylose can be used as an inexpensive carbon source for biotechnological applications. The model cyanobacterium *Synechocystis* sp. PCC 6803 lacks the ability to catabolize wood sugars as an energy source. Here, we generated four *Synechocystis* strains that heterologously expressed XylAB enzymes, which mediate xylose catabolism, either in combination with or without one of three xylose transporters, namely XylE, GalP, or Glf. Except for *glf*, which is derived from the bacterium *Zymomonas mobilis* ZM4, the heterologous genes were sourced from *Escherichia coli* K-12. All of the recombinant strains were able to utilize xylose in the absence of catabolite repression. When xylose was the lone source of organic carbon, strains possessing the XylE and Glf transporters were most efficient in terms of dry biomass production and xylose consumption and the strain lacking a heterologous transporter was the least efficient. However, in the presence of a xylose-glucose mixed sugar source, the strains exhibited similar levels of growth and xylose consumption. This study demonstrates that various bacterial xylose transporters can boost xylose catabolism in transgenic *Synechocystis* strains, and paves the way for the sustainable production of bio-compounds and green fuels from lignocellulosic biomass.

## Introduction

**FIGURE 1:**
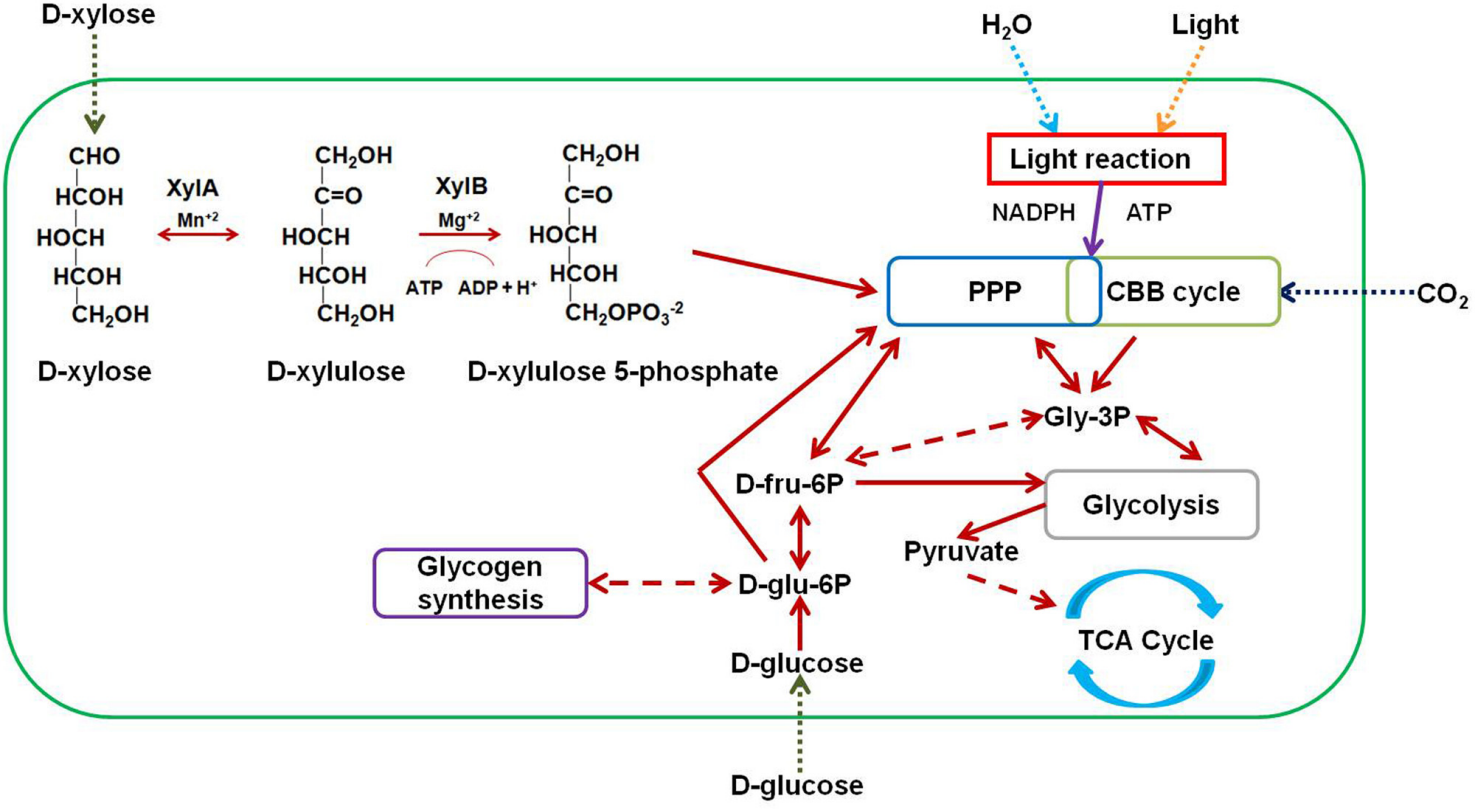
**Metabolic scheme for engineering *Synechocystis* strains.** D-xylose enters the transformant strains by means of native and heterologous transporters. Direction of entry of sugars, water, inorganic carbon (CO_2_), and light into the cell is shown as dotted arrows. D-xylose is converted to D-xylulose by D-xylose isomerase (XylA) in the presence of manganese ions (Mn^+2^). D-xylulose is further converted to D-xylulose 5-phosphate by the action of D-xylulokinase (XylB) in the presence of magnesium ions (Mg^+2^). D-xylulose 5-phosphate then enters the PPP. Bidirectional arrows indicate a reversible reaction or ability of an intermediate to enter and exit a pathway. Unidirectional arrows indicate a non-reversible reaction or the ability of an intermediate to either enter or exit a pathway. Dashed arrows indicate the involvement of one or more intermediates. Other abbreviations: CBB cycle, Calvin–Benson–Bassham cycle; ATP, adenosine triphosphate; NADPH, nicotinamide adenine dinucleotide phosphate; Gly-3P, glyceraldehyde 3-phosphate; D-fru-6P, D-fructose 6-phosphate; D-glu-6P, D-glucose 6-phosphate; TCA cycle, tricarboxylic acid cycle.

Lignocellulosic material is an abundant, inexpensive, and renewable source of carbon with potential industrial applications (Ragauskas et al., 2006). Large quantities of lignocellulosic biomass are generated in agricultural, forestry, and related industries each year. This residual biomass can be used to synthesize a number of value-added products (Pothiraj et al., 2006), especially energy-rich compounds that can be used as biofuels (Ragauskas et al., 2006; Lee and Lavoie, 2013; Anwar et al., 2014). Lignocellulose is typically composed of cellulose (40–50%), hemicellulose (20–30%), and lignin (10–15%) macromolecules bound together by hydrogen and covalent bonds (Malherbe and Cloete, 2002; Kumar et al., 2009). Hydrolysis of lignocellulose yields xylose, which is the second most abundant sugar in the biosphere after glucose. Xylose accounts for up to 35% of the total dry weight (DW) of plant materials (Gìrio et al., 2010). Catabolism of D-xylose begins with its transport into the cell by means of specific transporter proteins. Once inside the cell, xylose is isomerized by D-xylose isomerase (XylA) to yield D-xylulose, which in turn is irreversibly phosphorylated by the action of xylulokinase (XylB) to yield D-xylulose 5-phosphate, a pentose phosphate pathway (PPP) intermediate (**Figure 1**) (Doelle, 1975).

Few microorganisms are able to catabolize pentose sugars (Jojima et al., 2010). Even in pentose utilizing microorganisms, factors that impair pentose catabolism exist, such as catabolite repression (Vinuselvi et al., 2012), cellular redox imbalance, and insufficient transporter activities (Matsushika et al., 2009). Hence, effective utilization of pentose sugars by microorganisms requires the expression of heterologous genes (Jojima et al., 2010).

Although primarily photoautotrophic, a number of cyanobacteria can utilize organic carbon compounds (Eiler, 2006). In large-scale cyanobacterial cultures, mixotrophic growth conditions increase biomass production, whereas light and CO_2_ limit cyanobacterial growth and biosynthesis (Yao et al., 2014). Their unicellular nature, ability to photosynthesize and fix carbon dioxide, and amenability to genetic modification make cyanobacteria promising host organisms for biotechnological applications (Lu, 2010).

Several groups have introduced heterologous xylose transporter and/or metabolic genes into bacterial strains either to impart or improve the ability to transport and/or catabolize xylose. In an early study, the xylose catabolic genes *xylAB* and PPP genes *talB*-*tktA* from *Escherichia coli* were successfully expressed with the help of the GAP and ENO promoters, respectively, in *Zymomonas mobilis* CP4 to obtain a co-fermenting strain (Zhang et al., 1995). Similarly, the *E. coli xylAB* genes were successfully expressed under the *trc* promoter in the bacterium *Corynebacterium glutamicum* R to generate a diauxically growing strain (Kawaguchi et al., 2006), and also under the *tac* promoter in *Pseudomonas putida* S12. A laboratory evolution approach was used to generate a *P. putida* S12 strain with an increased biomass yield. The strain, however, failed to overcome diauxic growth. Introduction of the transporter genes *xylFGH* under the *tac* promoter failed to improve xylose metabolism, indicating that transport was not the limiting factor (Meijnen et al., 2008). *xylAB* genes from *Streptomyces lividans* TK23, when expressed under the *tac* promoter in the actinobacteria *Rhodococcus opacus* PD630 and *Rhodococcus jostii* RHA1, yielded efficient co-fermenting strains (Xiong et al., 2012). Heterologous expression of the Glf transporter from *Z*. *mobilis* CP4, which had been modified by error-prone PCR and random deletion/ligation, improved xylose transport in *E*. *coli* BL21 (Ren et al., 2009). One of the first reported attempts to engineer a cyanobacterium that could metabolize xylose involved *Synechococcus elongatus* PCC 7942. The wild-type strain appears to catabolize xylose slowly. Expression of the *E*. *coli xylE* transporter gene under the control of the *trc* promoter impaired growth due to the intracellular accumulation of xylose. However, introduction of the *xylEAB* operon from the same source organism under the control of the *trc* promoter doubled growth in the presence of xylose (McEwen et al., 2013). In another study, expression of the *E*. *coli xylAB* genes under the *psbA* promoter increased the rate of ethylene production in the presence of xylose. Furthermore, expression of the *E*. *coli xylAB* genes in a glycogen synthase mutant enhanced the synthesis of keto acids. Introduction of the *E*. *coli* XylFGH transporter enhanced xylose utilization only at concentrations of above 10 mM (Lee et al., 2015). In addition to bacteria, several yeast strains have been engineered for effective catabolism of xylose, with strategies ranging from expression of heterologous transporter-catabolic genes from bacteria and other fungi, to random mutagenesis-evolutionary engineering of the recombinant strains (Young et al., 2010).

In this study, we compared the biomass yield and xylose uptake of four recombinant *Synechocystis* sp. PCC 6803 (hereafter *Synechocystis*) strains heterologously expressing xylose-specific catabolic genes *xylAB* without or with one of three known xylose transporters (XylE, GalP, Glf). XylE (proton symporter), GalP (proton symporter), and Glf (uniporter) are members of the Major Facilitator Superfamily (hereafter MFS) class of transporter proteins (Jojima et al., 2010). The *glf* transporter gene is native to *Z*. *mobilis* ZM4, whereas the other heterologous genes used in this study were sourced from *E*. *coli* K-12. Here we report the production of recombinant *Synechocystis* strains with various abilities to utilize xylose in the absence of catabolite repression. We show that the ability of *Synechocystis* to utilize organic carbon sources can be enhanced by the heterologous expression of efficient transporters.

## Materials and Methods

### Bacterial Strains and Growth Conditions

*Escherichia coli* strain K-12 was used as a source organism for the amplification of xylose-specific transporter and catabolic genes. *E. coli* XL1-Blue (Stratagene) and TOP10 (Thermo Fisher Scientific) were used for DNA cloning and plasmid construction. The strains were grown at 37°C on solid LB medium or in liquid LB medium with shaking (220 rpm) in the presence of an appropriate antibiotic, if needed. Antibiotic concentrations used for selection were as follows: ampicillin (Fisher Scientific), 100 μg/ml for liquid and solid media; kanamycin (Fisher Scientific), 25 μg/ml for liquid and solid media; and spectinomycin (Fisher Scientific), 25 μg/ml for liquid medium and 50 μg/ml for solid medium.

*Zymomonas mobilis* ZM4 was used as a source organism for amplification of xylose transporter-specific gene. The strain was grown at 28°C on solid YP medium or in liquid YP medium (1% yeast extract, 2% Bacto-peptone) with shaking (220 rpm).

### Plasmid Construction

To construct the plasmids designed to insert xylose transporter genes into *Synechocystis* sp. PCC 6803, the kanamycin resistance cassette was spliced out from plasmid pUC4K (Vieira and Messing, 1982) using *Bam*HI and inserted into pBluescript II SK+ (Stratagene) digested with *Bam*HI. The neutral site sequence (near *slr1285*; hereafter referred to as neutral site 1) (Xue et al., 2014b) was amplified from genomic DNA isolated from *Synechocystis* as upstream and downstream regions using the following primers:

5′-ACTCGGTACCGGCAATGCAATTAATTAAAAATGG-3′ (forward primer) and 5′-ACTCCTCGAGTCTATTGTTGGAAGGTTGCTG-3′ (reverse primer) for the upstream region; and 5′-ACTCACTAGTGTGAAAAAATATTGACATTAAGATATC-3′ (forward primer) and 5′-ACTCCCGCGGGGAACCAGATTTTTAGGATG-3′ (reverse primer) for the downstream region. The upstream and downstream fragments were inserted between the *Kpn*I-*Xho*I sites and *Spe*I-*Sac*II sites of the modified pBluescript SK+ plasmid, respectively. Next, a ∼0.4-kb region encompassing the *psbA2* promoter (hereafter *psbA2* promoter) was amplified from *Synechocystis* genomic DNA using the following primers:

5′-ACTCGTCGACGGTATATGGATCATAATTGTATGC-3′ (forward primer) and 5′-ACTCGAATTCTTGGTTATAATTCCTTATGTATTTGTC-3′ (reverse primer). The amplified fragment was inserted between the *Sal*I-*Eco*RI sites of the plasmid. Then, the ∼0.5-kb 5ST1T2 double terminator region was amplified from the pBTac-1 plasmid (Boehringer Mannheim) using the following primers:

5′-ACTCCTGCAGCCAAGCTTGGCTGTTTTGG-3′ (forward primer) and 5′-ACTCGGATCCATTGAAGCATTTATCAGGGTTATTG-3′ (reverse primer). The fragment was inserted between the *Pst*I-*Bam*HI sites of the modified pBluescript II SK+ plasmid. Finally, the xylose transporter genes, *xylE*, *galP*, and *glf*, were amplified from genomic DNA isolated from *E. coli* K-12 (*xylE*, *galP)* or the *Z. mobilis* ZM4 strain (*glf)*. The primers used to amplify the transporter genes were as follows:

5′-ACTCGAATTCATGAATACCCAGTATAATTC-3′ (forward primer) and 5′-ACTCCTGCAGTTACAGCGTAGCAG-3′ (reverse primer) for *xylE*; 5′-ACTCGAATTCATGCCTGACGCTAA-3′ (forward primer) and 5′-ACTCCTGCAGTTAATCGTGAGCG-3′ (reverse primer) for *galP*; and 5′-ACTCGAATTCATGAGTTCTGAAAGTAGT-3′ (forward primer) and 5′-ACTCCTGCAGCTACTTCTGGGAG-3′ (reverse primer) for *glf*.

The amplicons were inserted between the *Eco*RI-*Pst*I sites of the plasmid to generate three individual plasmids.

To construct the plasmid designed to insert xylose catabolic genes, the spectinomycin resistance cassette was spliced from plasmid pHP45Ω (Prentki and Krisch, 1984) using *Bam*HI and inserted into the pBluescript II SK+ plasmid at the *Bam*HI site. The neutral site sequence (*slr0168*; hereafter called neutral site 2) (Kunert et al., 2000) was amplified from *Synechocystis* genomic DNA as upstream and downstream regions using the following primers:

5′-ACTCGGTACCATGACTATTCAATACACCC-3′ (forward primer) and 5′-ACTCGTCGACCACCTGCACCAGACCA-3′ (reverse primer) for the upstream region; and 5′-ACTCACTAGTTTGGGGCTGGCGGATT-3′ (forward primer) and 5′-ACTCCCGCGGCTAAGTCAGCGTAAATCTG-3′ (reverse primer) for the downstream region. The upstream and downstream fragments were inserted between the *Kpn*I-*Sal*I sites and *Spe*I-*Sac*II sites of the plasmid, respectively. The *psbA2* promoter and 5ST1T2 double terminator were amplified and cloned into the plasmid as described above. In the last step, the xylose catabolic genes *xylAB* were amplified from genomic DNA isolated from the *E. coli* K-12 strain using the following primers:

5′-ACTCGAATTCATGCAAGCCTATTTTGACCA-3′ (forward primer) and 5′-ACTCCCTGCAGGTTACGCCATTAATGG-3′ (reverse primer). The *xylAB* amplicon was inserted between the *Eco*RI-*Pst*I sites of the plasmid.

Note that, in the case of *xylAB*, only the region from the start codon of *xylA* to the end codon of *xylB* was amplified from the source organism; no other regulatory elements from the *xylAB* operon were included.

### *Synechocystis* Culture Conditions, Transformation, and Segregation

*Synechocystis* strains were grown on solid or in liquid BG-11 medium at 30°C under 50 μE m^-2^s^-1^ light intensity with shaking (200 rpm). When OD_730_ reached ∼0.8, transformations were carried out as described (Xue et al., 2014a). Once the presence of the heterologous insert was confirmed by PCR analysis, cells were streaked on BG-11 plates containing successively higher antibiotic concentrations followed by PCR tests to ensure complete segregation of the transformants.

### Reverse Transcription PCR

Total RNA was isolated from 80-ml cultures of *Synechocystis* strains possessing the *xylAB* genes at OD_730_ ∼0.7, as previously described (Mohamed and Jansson, 1989). A Turbo DNA-Free Kit (Thermo Fisher Scientific) was used to remove contaminating genomic DNA. The reverse transcription reactions were carried out using Superscript III enzyme (Thermo Fisher Scientific) and random primers (New England BioLabs). The cDNA molecules thus synthesized were then used as templates for PCR, employing the same set of primers used to amplify the heterologous transporter-catabolic genes. The same primer sets were employed to check the negative controls, in which DNase-treated RNA molecules were used as templates. *petA* (*sll1317*), which was used as a positive control, was amplified using the following primers:

5′-ACTCGAATTCATGAGAAACCCTGATACTTTGGGGCTGTGGACGAAAAC-3′ (forward primer) and 5′-ACTCCTGCAGCTAGAAATTAAGTTCGGCAGCTTGAACTTTTTCAATCTG-3′ (reverse primer). For a longer template, i.e., the *xylAB* genes, the SuperScript One-Step RT-PCR system for long templates (Thermo Fisher Scientific) was used as per manufacturer’s instructions. PCR products were analyzed on a 0.8% agarose gel.

### Cell Extract Preparation, SDS-PAGE, and Immunoblot Analysis

*Synechocystis* strains (wild-type and strains possessing the *xylAB* genes) were grown under the conditions described above, until OD_730_ reached 0.6–0.8. Whole cell extracts were obtained and 20 μg protein samples were electrophoresed as described (Xue et al., 2014a). After electrophoresis, gels were blotted onto nitrocellulose membranes using the Trans-Blot System (Bio-Rad). Membranes were probed with XylA- and XylB-specific primary antibodies of rabbit origin (raised by Dr. Qiang Wang) and goat anti-rabbit alkaline phosphatase-conjugated secondary antibodies (Sigma–Aldrich). Antigen-antibody interactions were visualized using the BCIP/NBT Kit (Thermo Fisher Scientific).

### Demonstration of ^14^C-Labelled D-Xylose Uptake

*Synechocystis* strains (wild-type and strains harboring the *xylAB* genes) were grown as described above, until the OD_730_ reached ∼0.8. Then, 10 ml of the cells were washed thrice with BG-11 medium and OD_730_ was adjusted to 0.6. Next, 1 ml of the cultures was grown in the presence of 400 nM ^14^C-labelled xylose [D-(1-^14^C)] (American Radiolabeled Chemicals) in 10 ml snap-capped glass tubes under similar growth conditions for 1 h. Nine milliliters of BG-11 medium was added to the cultures and they were filtered through a 0.45 μm pore-size nitrocellulose membrane (Bio-Rad). The membranes were washed thrice with 10 ml BG-11 medium, air dried, and suspended in 10 ml scintillation fluid (PerkinElmer) for 24 h. Radioactivity was measured using an LS 6500 scintillation counter (Beckman).

### Biomass Measurement

*Synechocystis* biomass was measured in terms of DW as previously described (Davies et al., 2014). Cultures of *Synechocystis* strains were initiated in 100 ml BG-11 liquid medium at 30°C with shaking (200 rpm) and an initial OD_730_ of 0.05. The cultures were provided with: for autotrophic growth, 50 μE m^-2^ s^-1^ light; for mixotrophic growth, 5 mM (750 mg/L) xylose and/or 5 mM glucose (900.8 mg/L) along with 50 μE m^-2^ s^-1^ light; for light-activated heterotrophic growth (hereafter LAHG) conditions (Anderson and McIntosh, 1991), 5 mM xylose (750 mg/L) and/or 5 mM glucose (900.8 mg/L) along with 50 μE m^-2^ s^-1^ light for 10 min per day; and for dark growth, neither sugar nor light. For the dark and LAHG cultures, biomass measurements were made every 24 h for 7 days, while for autotrophic and mixotrophic cultures, biomass values were estimated every 6 h for 3 days.

### Enzymatic Assays for Measurement of Sugar Uptake

Enzymatic uptake assays were performed for cultures grown in the presence of 5 mM xylose and 5 mM each of xylose and glucose. One milliliter of culture, collected at the time points specified for biomass measurements, was immediately filtered using 0.45 μm pore-size nylon membrane syringe filters (Fisher Scientific) to obtain cell-free media.

The amount of xylose present in the filtered medium fractions was measured using a D-Xylose Assay Kit (Megazyme) as per manufacturer’s instructions. The sequential action of xylose mutarotase and β-xylose dehydrogenase generates NADH molecules. The amount of NADH is stoichiometric with that of D-xylose and is calculated from the difference in OD_340_ values before and after β-xylose dehydrogenase action.

The amount of glucose present in the filtered medium fractions was measured using the D-Glucose Assay Kit-GOPOD Format (Megazyme) as per manufacturer’s instructions. Sequential action of glucose oxidase and peroxidase generates quinoneimine. The amount of dye formed is stoichiometric with that of D-glucose and was calculated from OD_510_ values obtained for the samples relative to the OD_510_ value obtained for a standard.

Data obtained from the biomass measurements and the enzymatic assays were used to calculate the sugar uptake rates by the *Synechocystis* strains during each time interval, using a previously described mathematical formula (Munyon and Merchant, 1959).

## Results

### Construction of *Synechocystis* Strains Possessing Heterologous Xylose-Specific Genes

**FIGURE 2:**
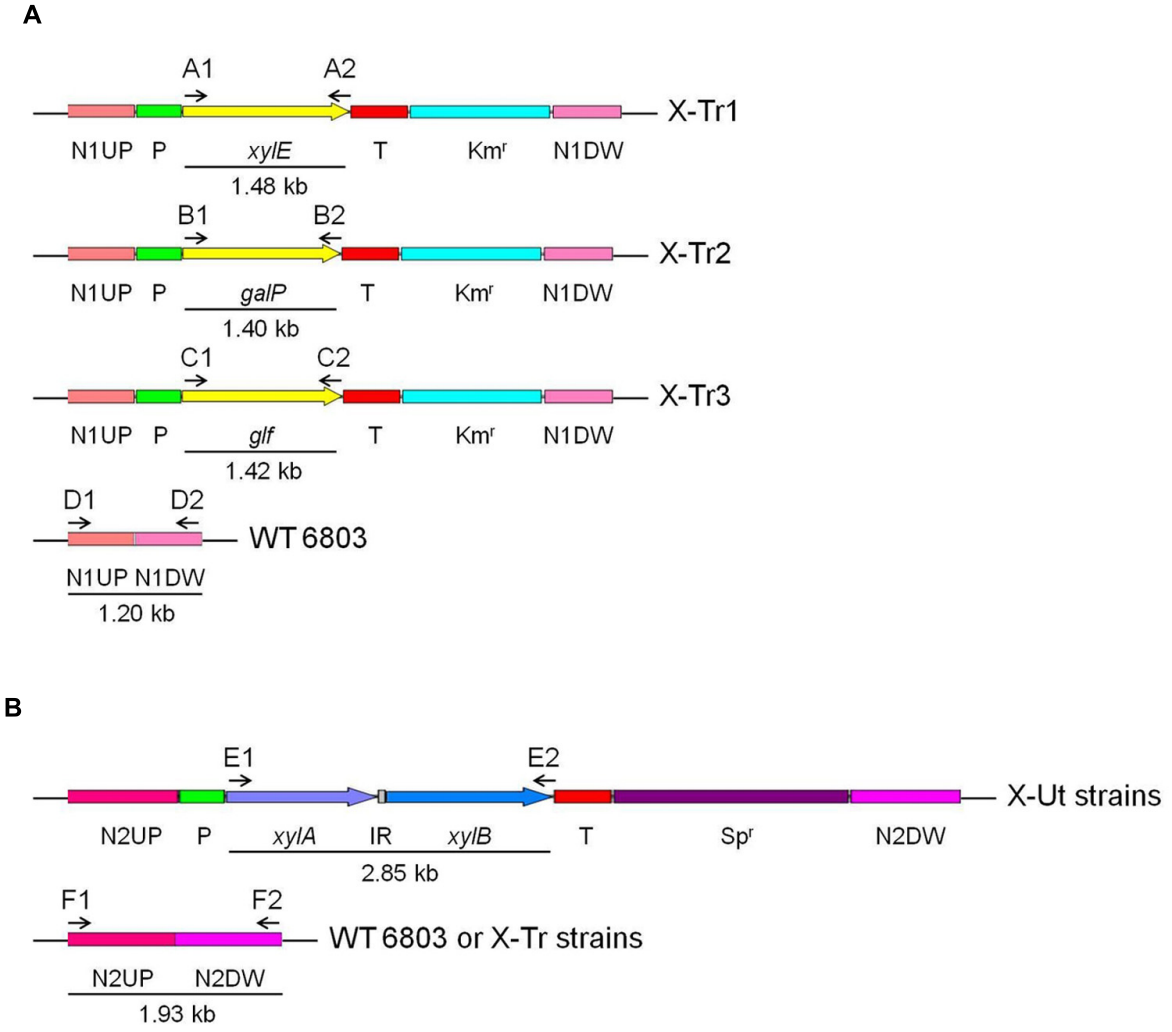
**Transformation of *Synechocystis* strains.** All transgenes used in the study were expressed under the control of the *psbA2* promoter (Prm) and 5ST1T2 terminator (Trm). Intergenic region (IR), if present between individual genes, is represented as a gray box. Numbered letters and arrows represent the primer sets used to insert genes and evaluate segregation, and the direction of the amplifications, respectively. **(A**) Genes encoding the xylose transporters were inserted into neutral site 1 in the wild-type *Synechocystis* genome using upstream (N1UP) and downstream (N1DW) regions for homologous recombination. The kanamycin resistance cassette (Km^r^) was used for selection and segregation of the transformants. **(B)** The *xylAB* genes, encoding enzymes that funnel xylose into the pentose phosphate pathway, were inserted into neutral site 2 in the genomes of *Synechocystis* strains carrying one of the three xylose transporter genes and the wild-type strain using upstream (N2UP) and downstream (N2DW) regions for homologous recombination. The spectinomycin resistance cassette (Sp^r^) was used for selection and segregation of the transformants.

*Synechocystis* strains possessing xylose transporter and catabolic genes were developed from a *Synechocystis* sp. PCC 6803 wild-type strain (hereafter WT). The construction process involved two rounds of transformation. In the first round, one of the three xylose transporter genes, *xylE* or *galP* from *E*. *coli* K-12 or *glf* from *Z*. *mobilis* ZM4, was introduced into neutral site 1 (Xue et al., 2014b) in WT *Synechocystis* via homologous recombination to generate three individual strains, X-Tr1, X-Tr2, and X-Tr3, respectively (**Table 1**). The plasmid constructs designed for integration of the transporter genes included the *psbA2* promoter of *Synechocystis* origin, 5ST1T2 double terminator of *E*. *coli* origin, kanamycin resistance cassette, neutral site 1 divided into ∼600 bp upstream and ∼600 bp downstream regions, and one of the three aforementioned transporter genes (**Figure 2A**).

**Table 1:** Synechocystis strains used in this study.

Strain	Genotype	Description
WT	Wild-type *Synechocystis* sp. 6803	Wild-type genomic sequence
X-Tr1	*xylE*-Δ*Neu 1*	*xylE* inserted at neutral site 1 (near *slr1285*)
X-Tr2	*galP*-Δ*Neu 1*	*galP* inserted at neutral site 1 (near *slr1285*)
X-Tr3	*glf*-Δ*Neu 1*	*glf* inserted at neutral site 1 (near *slr1285*)
X-Ut1	*xylE*-Δ*Neu 1*::*xylAB*-Δ*Neu 2*	*xylE* inserted at neutral site 1 (near *slr1285*), *xylAB* genes inserted at neutral site 2 (*slr0168*)
X-Ut2	*galP*-Δ*Neu 1*::*xylAB*-Δ*Neu 2*	*galP* inserted at neutral site 1 (near *slr1285*), *xylAB* genes inserted at neutral site 2 (*slr0168*)
X-Ut3	*glf*-Δ*Neu 1*::*xylAB*-Δ*Neu 2*	*glf* inserted at neutral site 1 (near *slr1285*), *xylAB* genes inserted at neutral site 2 (*slr0168*)
X-Ut4	*xylAB*-Δ*Neu 2*	*xylAB* genes inserted at neutral site 2 (*slr0168*)

In the second round of transformation, the *xylAB* genes, which are responsible for funneling xylose into the PPP, were introduced into neutral site 2 (Kunert et al., 2000) in the three *Synechocystis* strains generated in the first round of transformation as well as in the WT strain via homologous recombination to obtain four resultant strains, X-Ut1, X-Ut2, X-Ut3, and X-Ut4 (**Table 1**). The plasmid construct designed to integrate the xylose catabolic genes included the same promoter and transcriptional terminator as used in the transporter gene-specific plasmid constructs, the spectinomycin resistance cassette, neutral site 2 divided into ∼970 bp upstream and ∼970 bp downstream regions, and *xylAB* genes (**Figure 2B**). The *xylAB* genes consist of two individual genes, *xylA* (encoding D-xylose isomerase) and *xylB* (encoding D-xylulokinase), along with the ribosomal binding site (hereafter RBS) present between them.

We used the native *psbA2* promoter to drive expression of both the transporter genes and the catabolic genes. *psbA2* is a strong, light-sensitive promoter that enhances gene expression under high light conditions (Mohamed and Jansson, 1989) and regulates the expression of heterologous genes in response to variations in light intensity (Lindberg et al., 2010). This promoter has been used to express a number of heterologous genes (Zhou et al., 2014).

### Insertion of Heterologous Genes and Segregation of the Transformant Strains

The transporter as well as xylose utilization-specific heterologous genes were introduced into the neutral sites of the *Synechocystis* genome by homologous recombination. After each round of transformation, putative transformants were checked for the presence of heterologous gene inserts. Cells from one of the transformant colonies were cultivated successively under increasing antibiotic pressure to achieve complete segregation, in which selection of only those cells that had acquired all the genome copies carrying the insert was ensured. Only after confirmation of segregation, were strains used for the next round of transformation and further work.

**FIGURE 3:**
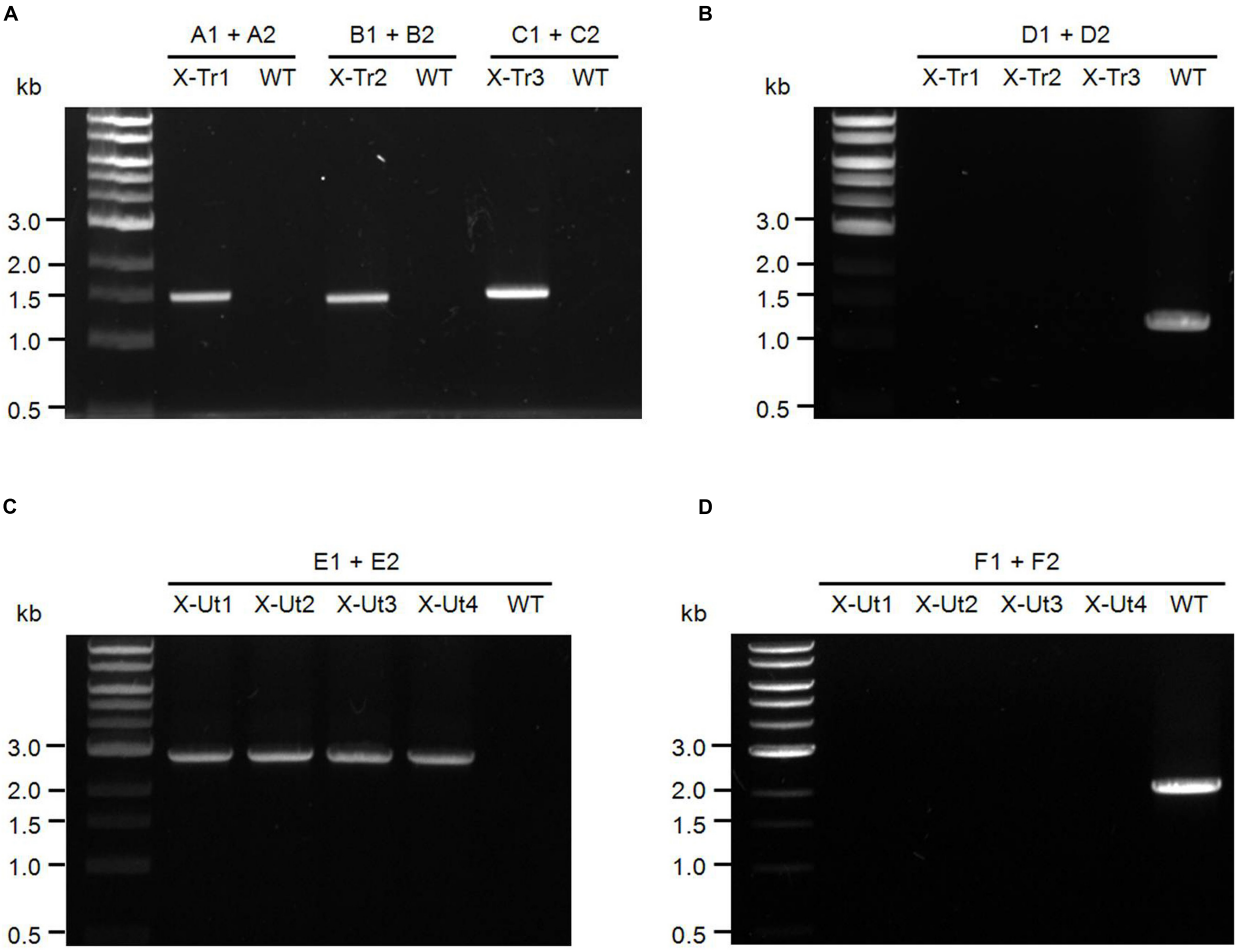
**Test of insertion of heterologous genes and chromosomal segregation for *Synechocystis* strains. (A)** Primers upstream and downstream of the xylose transporter genes x*ylE* (A1–A2), *galP* (B1–B2), and *glf* (C1–C2) were used to confirm the presence of the inserts in the respective transformants (**Figure 2A**) and also in the WT strain as the negative control. In the presence of the templates, primers A1–A2, B1–B2, and C1–C2 produced fragments of ∼1.48, ∼1.40, and ∼1.42 kb, respectively. **(B)** Primers upstream and downstream of intact neutral site 1 (D1–D2) were used to examine the chromosomal segregation for respective transformants (**Figure 2A**) along with the WT strain as a positive control. In the absence of uninterrupted neutral site 1, primers D1–D2 failed to produce PCR products in the transformants, whereas the WT strain yielded a ∼1.20-kb product. **(C)** Primers upstream and downstream of the xylose catabolic genes *xylAB* (E1–E2) were used to examine the insert in respective transformants (**Figure 2B**) and also in the WT strain as the negative control. In the presence of the template, primers E1–E2 produced a product of ∼2.85 kb. **(D)** Primers upstream and downstream of intact neutral site 2 (F1–F2) were used to check the chromosomal segregation for respective transformants (**Figure 2B**) along with the WT strain as the positive control. In the absence of uninterrupted neutral site 2, primers F1–F2 failed to produce PCR products in the transformants, whereas the WT strain yielded a ∼1.93-kb product.

For the PCR analyses represented in **Figures 3A,B**, the three strains generated after the first round of transformation, i.e., X-Tr1, X-Tr2, and X-Tr3, were used. To verify the presence of transporter-specific heterologous genes, the same sets of primers used to amplify the respective genes from genomic DNA of source organisms were employed (A1/A2 for *xylE*, B1/B2 for *galP*, and C1/C2 for *glf*, **Figure 2A**), which generated products of approximately 1.48, 1.40, and 1.42 kb in length, respectively (**Figure 3A**). To verify segregation, the forward primer used to amplify the upstream region of neutral site 1 and the reverse primer used to amplify the downstream region of neutral site 1 were employed (D1/D2, **Figure 2A**), and extension times in the PCR cycles were set specifically to amplify the wild-type (uninterrupted) neutral site 1. PCR amplifications yielded a ∼1.20-kb band representing neutral site 1 in the WT strain, but failed to generate any product for the transformants, indicating the absence of uninterrupted neutral site 1 and hence complete segregation of the strains (**Figure 3B**).

To test for the presence of the catabolic genes *xylAB* in the strains obtained after the second round of transformation, i.e., X-Ut1, X-Ut2, X-Ut3, and X-Ut4, the same set of primers used to amplify the genes from genomic DNA of the source organism was employed (E1/E2, **Figure 2B**), which generated products of approximately 2.85 kb in length (**Figure 3C**). To verify the segregation with respect to neutral site 2, the forward primer used for amplification of the upstream region of neutral site 2 and the reverse primer used for amplification of the downstream region of neutral site 2 were employed (F1/F2, **Figure 2B**), and extension times in the PCR were set specifically to amplify wild-type neutral site 2. PCR amplifications yielded a ∼1.93-kb band representing neutral site 2 in the WT strain, but failed to generate any product for the transformants, indicating the absence of uninterrupted neutral site 2 and hence complete segregation of the strains (**Figure 3D**).

### Expression of Heterologous Genes at the Transcriptional Level

Once the segregation of the strains obtained after the second round of transformation was confirmed, transcription of the heterologous genes for the transporters and the catabolic genes in the X-Ut1, X-Ut2, X-Ut3, and X-Ut4 strains was examined by RT-PCR. For the expression studies, both a positive and negative control were included. For the positive control, expression of *petA* (*sll1317*), which encodes apocytochrome *f*, a core subunit of the cytochrome *b_6_f* complex, was tested. As a negative control for each strain, we examined whether the corresponding heterologous genes could be amplified from DNase-treated RNA samples obtained from each strain by PCR, to confirm the absence of any leftover DNA in the RNA samples.

**FIGURE 4:**
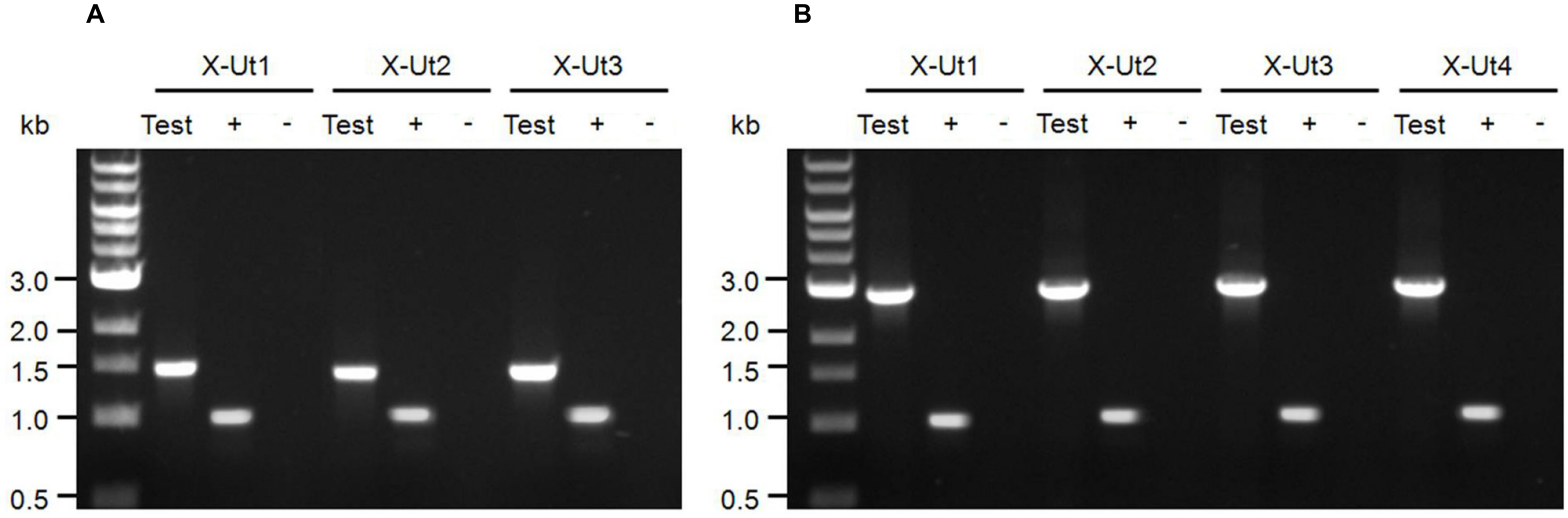
**Transcription evaluation of xylose-specific genes. (A)** Primers upstream and downstream of the xylose transporter genes *xylE* (A1–A2), *galP* (B1–B2), and *glf* (C1–C2) were used (**Figure 2A**) to examine whether these genes were transcribed in the corresponding strains represented as ‘Test.’ In the presence of the templates, primers A1–A2, B1–B2, and C1–C2 produced products of ∼1.48, ∼1.40, and ∼1.42 kb in length, respectively. **(B)** Primers upstream and downstream of the xylose catabolic genes *xylAB* (E1–E2) were used (**Figure 2B**) to evaluate the transcription of the genes in the corresponding strains represented as ‘Test.’ In the presence of the templates, primers E1–E2 produced products of ∼2.85 kb. For the positive control (+), transcription of *petA* was examined using a gene-specific primer pair. The primer set produced a ∼0.99-kb product from cDNA templates of the transformants. For the negative control (-), amplification of heterologous genes from the corresponding RNA samples was evaluated using the gene-specific primer pairs. The primer pairs failed to generate any PCR product, indicating the absence of DNA contamination in the RNA samples.

When cDNA samples from the aforementioned strains were subjected to PCR using the same primer sets used to confirm gene insertion, products of the expected size were amplified. A ∼0.98-kb band was present for all positive controls and heterologous gene-specific bands were absent in the negative controls for all strains (**Figures 4A,B**). These results indicate the successful transcription of all the heterologous genes and corroborate the quality of the RNA samples.

### Expression of the Catabolic Genes at the Translational Level

**FIGURE 5:**
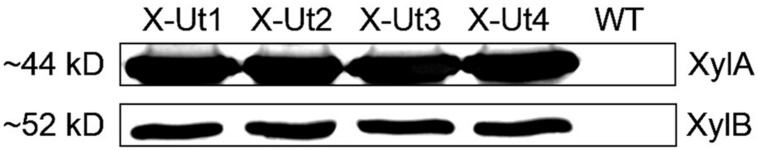
**Immunodetection of xylose catabolic proteins.** Protein samples extracted from *Synechocystis* transformant strains, X-Ut1, X-Ut2, X-Ut3, and X-Ut4 and the WT strain as the negative control were probed with specific polyclonal antibodies for the XylA (D-xylose isomerase) and XylB (D-xylulokinase) enzymes. Each lane was loaded with 20 μg of total protein fraction. The expected molecular masses for XylA and XylB proteins are indicated.

To study the expression of the xylose catabolic genes, *xylA* and *xylB*, at the protein level, specific polyclonal antibodies were raised by Dr. Qiang Wang (Institute of Hydrobiology, Wuhan, China) against XylA and XylB proteins overexpressed in *E. coli*. The antibodies were used to detect the presence of the proteins in the total cell extracts obtained from the X-Ut1, X-Ut2, X-Ut3, and X-Ut4 strains, and the WT strain was used as the negative control. XylA protein, with a molecular weight of ∼44 kD (Schellenberg et al., 1984), was present in the total cell extracts obtained from the transformants. Similarly, the presence of XylB protein, with an estimated molecular weight of ∼52 kD (Lawlis et al., 1984), was detected in the cell extracts isolated from the transformant strains (**Figure 5**). Expression of XylB showed that the RBS present between *xylA* and *xylB* of *E*. *coli* origin functioned in *Synechocystis*. Antibodies probing both of these proteins showed no cross-reaction with proteins present in the WT cell extract.

### Uptake Assay of D-[^14^C] Xylose as a Demonstration of Transporter Activity

**FIGURE 6:**
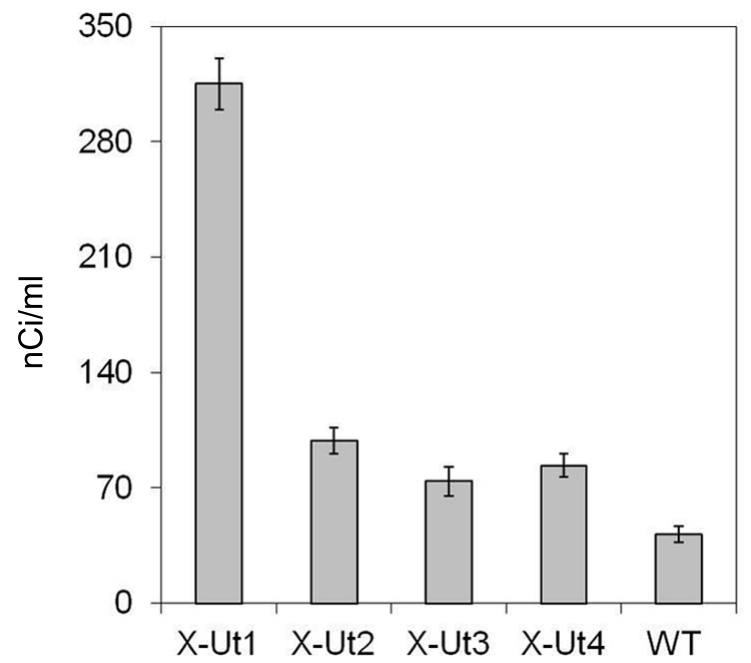
**Demonstration of D-[^14^C] xylose uptake.** To demonstrate the uptake of xylose, cultures of *Synechocystis* transformant strains, X-Ut1, X-Ut2, X-Ut3, and X-Ut4, and the WT strain at an OD_730_ of 0.6 were grown in the presence of D-[^14^C] xylose. Radioactivity detected in the cultures is represented in nCi/ml. Data were collected from three biological replicates and presented as means ± standard deviations.

To probe the activity of xylose transporter proteins in *Synechocystis*, we tested the ability of the X-Ut1, X-Ut2, X-Ut3, and X-Ut4 strain and also of the WT strain to take up D-[^14^C] xylose. At the end of a 1-h incubation period, the strains showed various degrees of radioactivity. The levels of radioactivity in X-Ut1, X-Ut2, X-Ut3, and X-Ut4 were ∼653%, ∼136%, ∼77%, and ∼100% greater than those observed in the WT strain (**Figure 6**). Thus, the transformants were better able to take up and use xylose than was the WT.

### Study of Biomass Accumulation Under Various Conditions

To study and compare the growth patterns exhibited by the strains generated after the first and second transformation events and the WT strain, we cultured the strains under different conditions (described in Materials and Methods). Biomass, i.e., DW, measurements were taken at 24-h intervals for 7 days in the case of dark and light-activated heterotrophic growth (LAHG) conditions and at 6-h intervals for 3 days for autotrophy and mixotrophy. In the absence of xylose, biomass yields were similar for all the strains. None of the strains were able to grow in darkness (data not shown). Under LAHG conditions in the presence of 5 mM glucose (hereafter LAHG-glucose conditions), at the end of the seventh day, the strains showed biomass yields of 0.329 ± 0.010 g/L. Under autotrophy and mixotrophy in the presence of 5 mM glucose (hereafter mixotrophy-glucose conditions), at the end of third day, the biomass yields obtained for all strains were 0.244 ± 0.015 g/L and 0.993 ± 0.013 g/L, respectively.

**FIGURE 7:**
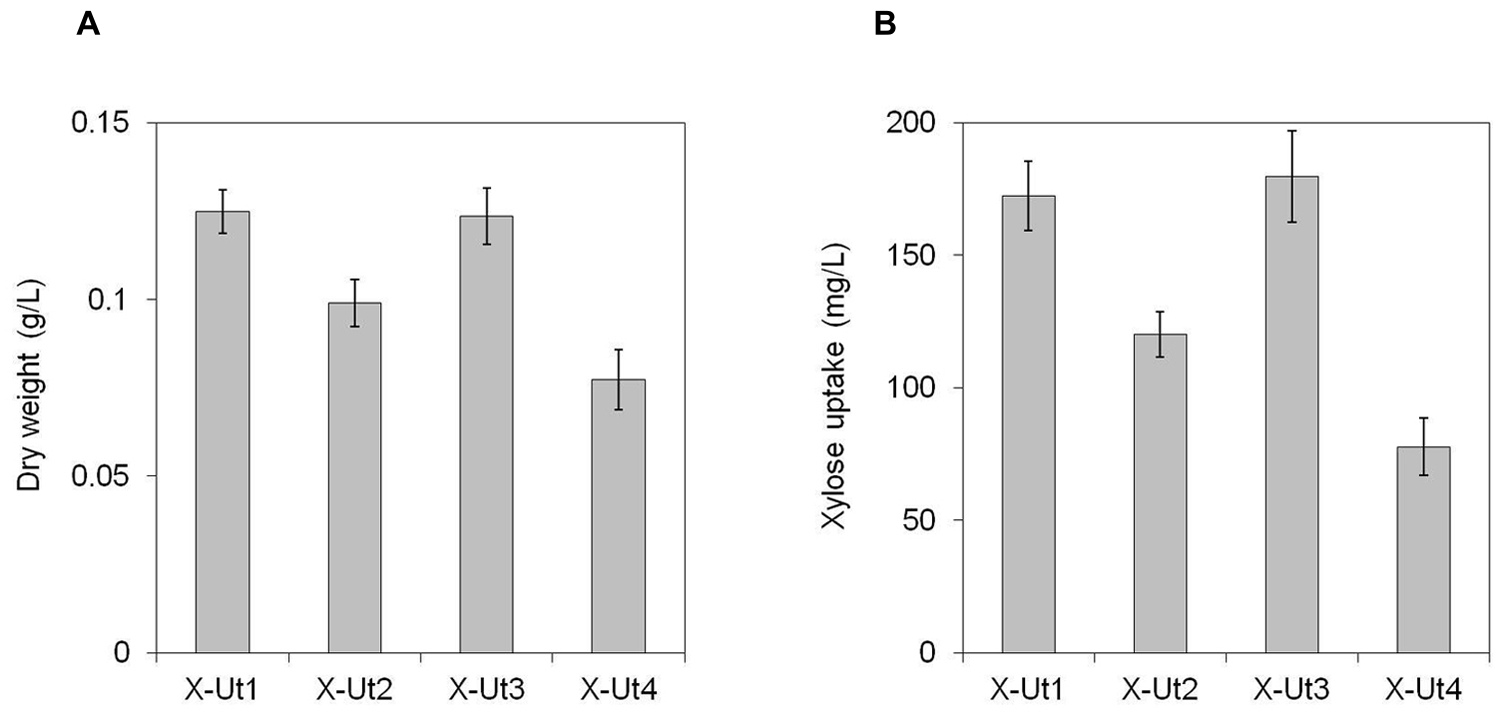
**Growth and xylose consumption under LAHG conditions in the presence of 5 mM xylose.** *Synechocystis* strains X-Ut1, X-Ut2, X-Ut3, and X-Ut4 were grown in the presence of 5 mM (750 mg/L) xylose. **(A)** Dry biomass values estimated at the end of the seventh day. **(B)** Xylose consumption at the end of the seventh day exhibited by the transformant strains, determined from the measurement of residual xylose in the cell-free medium. Data were collected from three biological replicates and are presented as means ± standard deviations.

**FIGURE 8:**
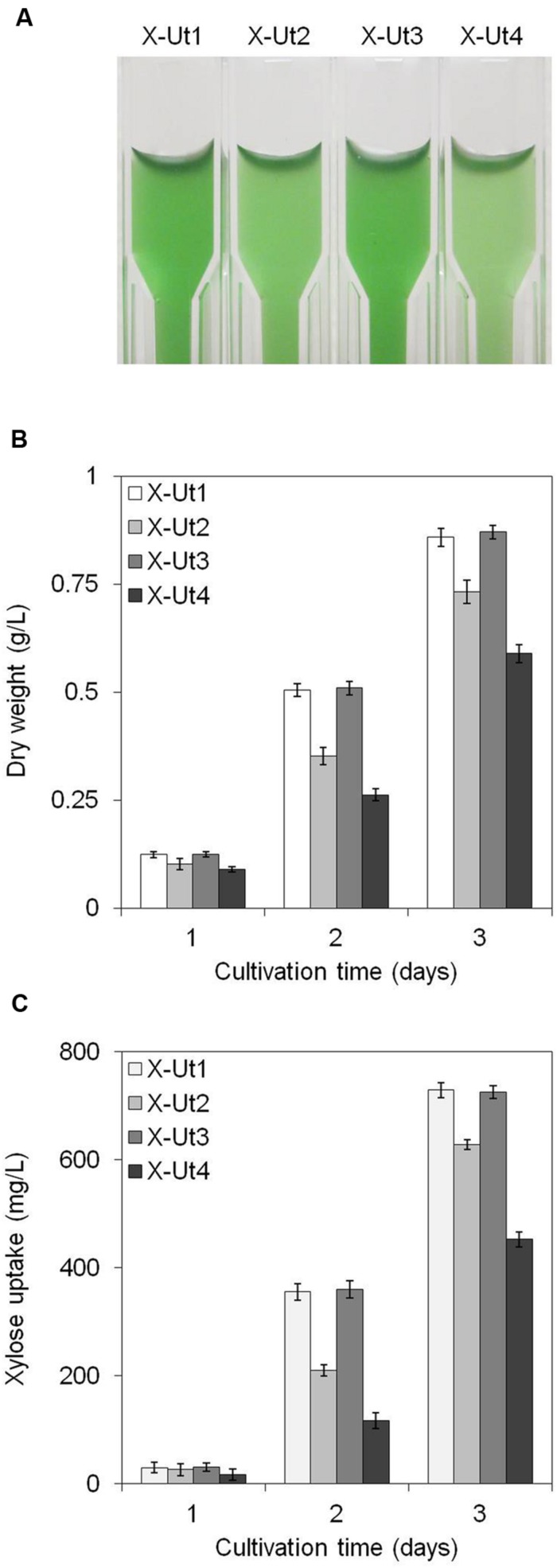
**Growth and xylose consumption under mixotrophy in the presence of 5 mM xylose.** *Synechocystis* strains X-Ut1, X-Ut2, X-Ut3, and X-Ut4 were grown in the presence of 5 mM (750 mg/L) xylose. **(A)** Photograph depicting differential growth shown by the transformant strains at the end of second day of culture. **(B)** Dry biomass values estimated at the end of first, second, and third days. **(C)** Xylose consumption at the end of the first, second, and third day exhibited by the transformant strains, determined from the measurement of residual xylose in filtered cell-free medium. Data represented in graphs were collected from three biological replicates and presented as means ± standard deviations.

Under LAHG conditions in the presence of 5 mM xylose (hereafter LAHG-xylose conditions), only X-Ut1, X-Ut2, X-Ut3, and X-Ut4 strains exhibited growth (data not shown for WT, X-Tr1, X-Tr2, and X-Tr3; **Figure 7A**). Under mixotrophy conditions in the presence of 5 mM xylose (hereafter mixotrophy-xylose conditions), the WT, X-Tr1, X-Tr2, and X-Tr3 strains showed biomass yields of 0.331 ± 0.014 g/L, which were similar to those obtained when grown under autotrophy, indicating the inability of the strains to utilize xylose. However, the X-Ut1, X-Ut2, X-Ut3, and X-Ut4 strains showed greater biomass yields under mixotrophy-xylose conditions than under autotrophy (**Figures 8A,B**). Biomass accumulation results demonstrate that all four of the transformants carrying the catabolic genes, namely X-Ut1, X-Ut2, X-Ut3, and X-Ut4, utilize xylose. After 7 days of culture under LAHG-xylose conditions, the X-Ut1, X-Ut2, and X-Ut3 strains showed ∼62, 28, and ∼60% greater biomass yields than did X-Ut4, respectively (**Figure 7A**). Under mixotrophy-xylose conditions, the strains carrying heterologous transporters showed higher biomass values at each time point. Notably, at the end of the second day of culture, X-Ut1, X-Ut2, and X-Ut3 strains showed ∼92, ∼34, and ∼94% greater biomass values than did the X-Ut4 strain (**Figure 8B**). For both LAHG-xylose and mixotrophy-xylose conditions, biomass measurement data for X-Ut1, X-Ut2, X-Ut3, and X-Ut4 strains at each time point are presented in Supplementary Figures S1A and S2A.

**FIGURE 9:**
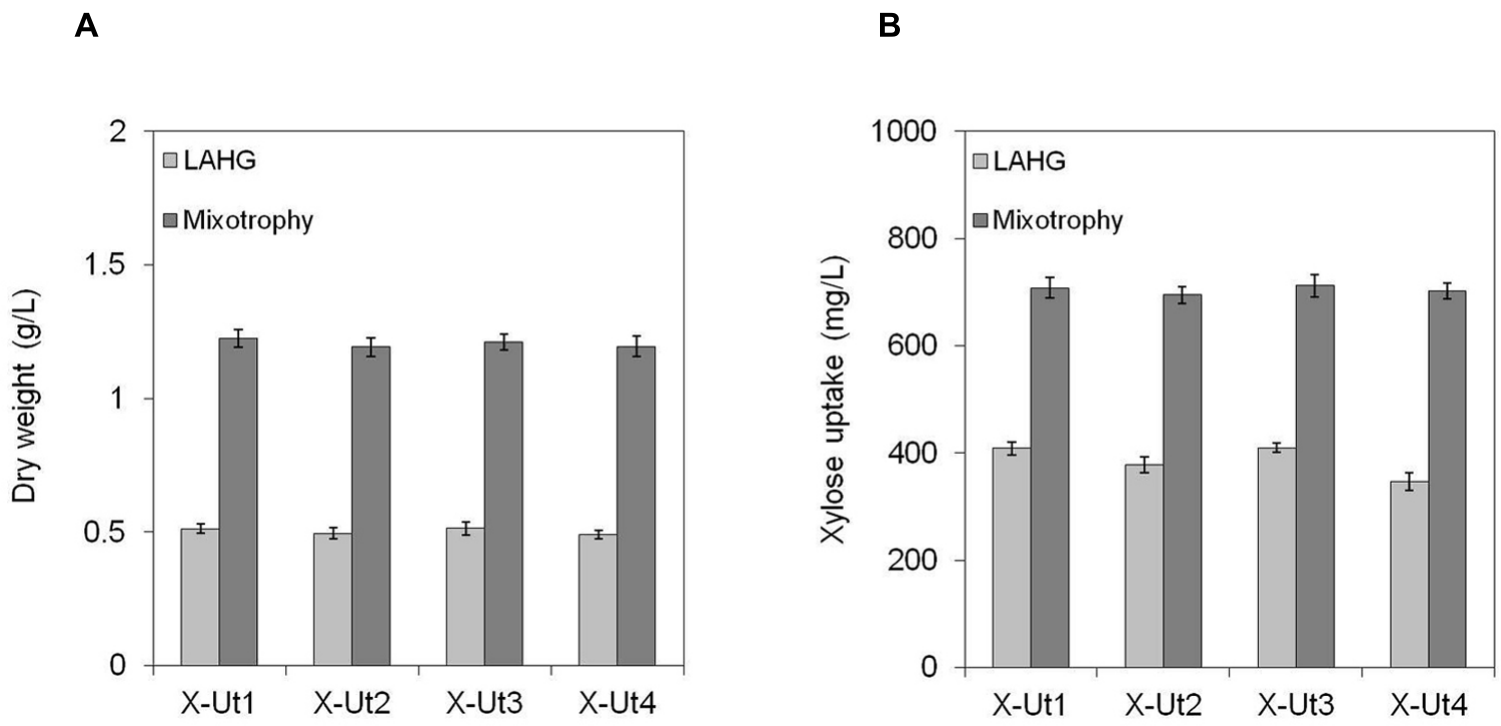
**Growth and xylose consumption in the presence of 5 mM xylose and glucose.** *Synechocystis* strains X-Ut1, X-Ut2, X-Ut3, and X-Ut4 were grown in the presence of 5 mM (750 mg/L) xylose and 5 mM (900.8 mg/L) glucose. **(A)** Dry biomass values estimated at the end of the seventh day for the LAHG condition and at the end of the third day for mixotrophy. **(B)** Xylose consumption at the end of the seventh day for the LAHG condition and at the end of the third day for mixotrophy, determined by the measurement of residual xylose in the cell-free medium. Data were collected from three biological replicates and presented as means ± standard deviations.

To examine the ability of the xylose consuming strains to grow in the presence of xylose and glucose, we cultured the strains under LAHG and mixotrophic conditions in medium supplemented with 5 mM each of xylose and glucose (hereafter LAHG-mixed sugar and mixotrophy-mixed sugar conditions). The xylose consuming strains showed similar biomass yields under both conditions, in contrast to the variable yields exhibited in the presence of xylose alone (**Figures 7A**, **8A,B** and **9A**). The absence of two distinct growth phases and the simultaneous uptake of both the sugars did not indicate diauxie in these strains (data not shown).

### Sugar Uptake Assays

**FIGURE 10:**
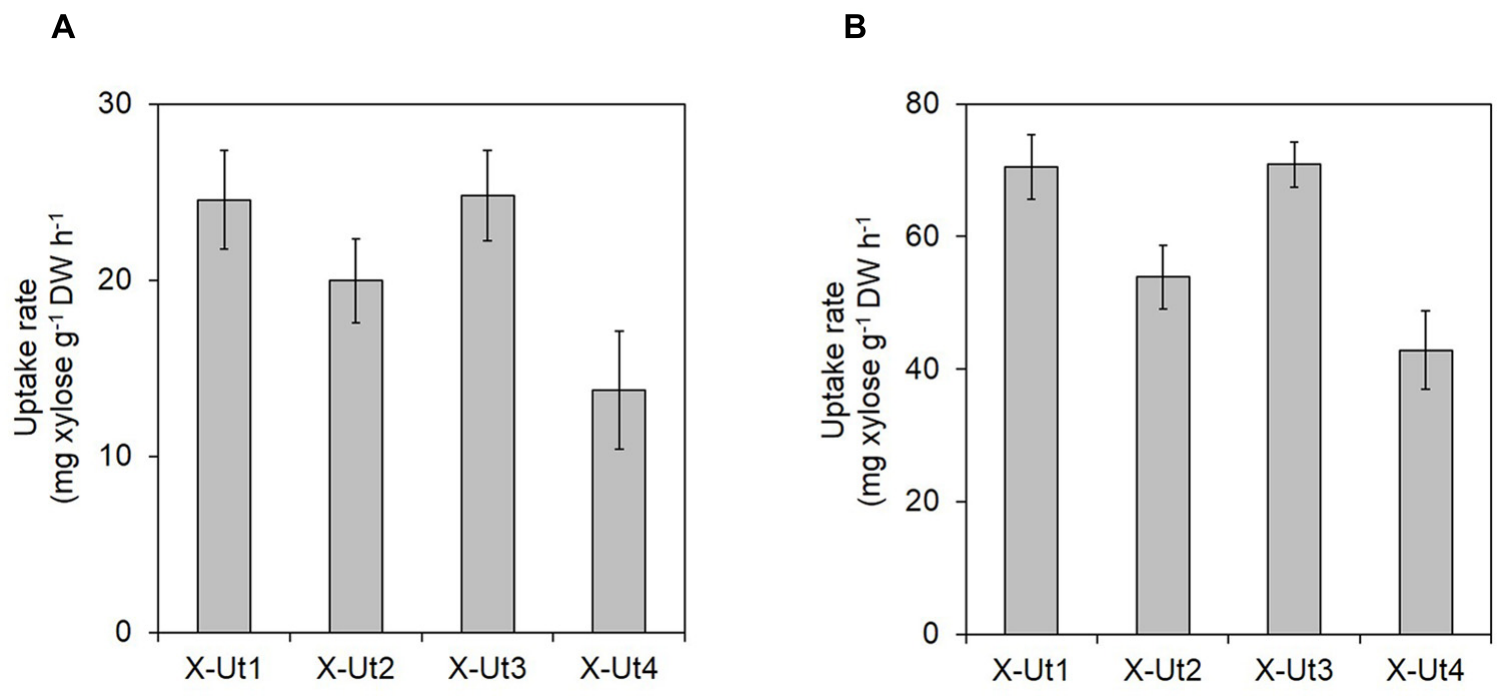
**Maximum xylose uptake rates in the presence of 5 mM xylose.** *Synechocystis* strains X-Ut1, X-Ut2, X-Ut3, and X-Ut4, were grown in the presence of 5 mM (750 mg/L) xylose. Maximum xylose uptake rates [mg xylose g^-1^ dry weight (DW) h^-1^] under **(A)** LAHG and **(B)** mixotrophy conditions were calculated using a previously described formula (Munyon and Merchant, 1959). For all the strains, maximum xylose uptake rates were observed between the sixth and seventh day under LAHG. Under mixotrophy, maximum xylose uptake rates were observed between the 42 and 48 h (for X-Ut1, X-Ut3, and X-Ut4) and the 48 and 54 h (for X-Ut2). Raw data were collected from three biological replicates. Uptake rates are presented as means ± standard deviations.

To measure xylose uptake by the strains generated after each transformation event and by the WT strain, we first cultured the strains under LAHG-xylose and mixotrophy-xylose conditions. The WT, X-Tr1, X-Tr2, and X-Tr3 strains that lacked the catabolic genes *xylAB* showed no change in the amount of residual xylose in the medium at the tested time points, indicating their inability to utilize xylose (data not shown). On the other hand, the X-Ut1, X-Ut2, X-Ut3, and X-Ut4 strains that possessed the *xylAB* genes showed varying degrees of xylose consumption under both growth conditions (**Figures 7B** and **8C**). After 7 days of culture under LAHG-xylose conditions, xylose consumption was ∼122, 55, and ∼131% greater in the X-Ut1, X-Ut2, and X-Ut3 strains, respectively, than in X-Ut4 (**Figure 7B**). Under the same conditions, the maximum xylose uptake rates exhibited by the X-Ut1, X-Ut2, and X-Ut3 strains were ∼78, ∼45, and ∼80% higher than that exhibited by X-Ut4 (**Figure 10A**). Under mixotrophy-xylose conditions, the strains carrying heterologous transporters showed higher xylose uptake than did the strain relying only on endogenous transporter/s at each time point. Notably, at the end of the second day of culture, the X-Ut1, X-Ut2, and X-Ut3 strains showed ∼205, ∼80, and ∼209% higher levels of xylose uptake than did X-Ut4 (**Figure 8C**). Under the same conditions, the maximum xylose uptake rates exhibited by the X-Ut1, X-Ut2, and X-Ut3 strains were ∼64, ∼26, and ∼65% higher than that exhibited by X-Ut4 (**Figure 10B**). For both LAHG-xylose and mixotrophy-xylose conditions, the sugar uptake data at each time point are presented in Supplementary Figures S1B and S2B.

When grown under LAHG and mixotrophy-mixed sugar conditions, the xylose consuming strains, X-Ut1, X-Ut2, X-Ut3, and X-Ut4, consumed similar amounts of xylose (**Figure 9B**), in accordance with the biomass accumulation data. Under LAHG-mixed sugar conditions, all of the glucose was consumed by the end of the sixth day, while under mixotrophy-mixed sugar conditions, all of the glucose was consumed by the end of 48 h (data not shown). In the presence of mixed sugars, the maximum sugar uptake rates exhibited by the xylose consuming strains were very similar, in contrast to the results obtained when xylose was the only organic carbon source. Also, a sharp reduction in the maximum xylose uptake rates was observed when glucose was present along with xylose compared to when xylose was the sole sugar, with the exception of the X-Ut4 strain, which exhibited similar maximum xylose uptake rates under LAHG-xylose and LAHG-mixed sugar conditions (**Figures 10A,B**; Supplementary Table S1).

When xylose was the sole organic carbon source, the X-Ut1 and X-Ut3 strains were the most efficient in terms of biomass accumulation and xylose uptake, X-Ut2 was intermediately efficient, and X-Ut4 was the least efficient. When glucose was used in addition to xylose, no significant difference was observed in the growth and xylose consumption patterns among the xylose consuming strains, in the defined period of study. These results demonstrate that heterologous expression of XylE, GalP, and Glf enhances xylose transport in *Synechocystis*, which results in more organic carbon being available for growth and metabolism via the engineered isomerase catabolic pathway.

## Discussion

In this study, we sought to construct and compare *Synechocystis* strains that were able to utilize xylose by the heterologous expression of genes native to *E*. *coli* and *Z*. *mobilis*. Since *Synechocystis* is a mesophilic Gram negative cyanobacterium, heterologous genes derived from other Gram-negative mesophiles, especially the ones encoding the transporters, were expected to function in the host strain. *E*. *coli* is naturally capable of utilization of xylose and its genes involved in the xylose metabolism have been used to engineer other bacteria and yeasts (Zhang et al., 1995; Kawaguchi et al., 2006; Meijnen et al., 2008; Young et al., 2010; Xiong et al., 2012; McEwen et al., 2013; Lee et al., 2015). Although *Z*. *mobilis* is not a natural xylose utilizer, its glucose transporter, Glf is able to take up xylose very efficiently and has been functionally expressed in *E*. *coli* in either modified or unmodified form (Weisser et al., 1995; Chen et al., 2009; Ren et al., 2009). Hence, *E*. *coli* and *Z*. *mobilis* were chosen as the source organisms for xylose specific genes in the study. Although *Synechocystis* was considered to lack xylose transporters, we independently, alongside another recent study (Lee et al., 2015), found that expression of the *xylAB* genes alone was sufficient to generate lines that could utilize xylose (**Figures 7A,B** and **8A–C**), indicating the involvement of endogenous sugar transporter/s. This also indicates that the xylose uptake observed in strains possessing non-native transporters was due to activities of both endogenous and heterologous transporters. GlcP (*sll0771*) is the only known MFS-type glucose transporter identified in *Synechocystis* (Lee et al., 2015), and this transporter shows an affinity for fructose as well (Zhang et al., 1989). Two of the heterologous MFS-type transporter proteins used in the study have also been shown to possess specificity for more than one substrate. The proton symporter GalP of *E*. *coli* origin, which is primarily a galactose transporter, is able to transport an array of other substrates, including xylose (Baldwin and Henderson, 1989). Similarly, the uniporter Glf of *Z*. *mobilis* origin, which is primarily a glucose transporter, is also able to transport fructose and xylose (Saier and Crasnier, 1996).

Our results either directly or indirectly show that we successfully expressed the xylose catabolic genes *xylAB* in *Synechocystis* (**Figures 5**, **6**, **7A,B** and **8A–C**), indicating that the *E*. *coli* RBS functions in *Synechocystis*. Heterologously expressed *E*. *coli xylAB* and *xylFGH* genes, which encode xylose catabolic enzymes and an ATP-binding cassette (ABC)-type transporter and contain unmodified RBSs, were recently shown to be involved in xylose transport and catabolism in engineered *Synechocystis* (Lee et al., 2015). In this work, we have compared the performance of *Synechocystis* strains engineered to catabolize xylose, relying on the native, non-specific xylose transporter/s with or without one of the three heterologous MFS type transporter proteins. With this strategy, we attempted to compare the performance of heterologous transporter proteins when expressed individually in *Synechocystis*, under LAHG and mixotrophic conditions. Unlike some other cyanobacterial species, *Synechocystis* is unable to grow heterotrophically under complete darkness, and requires brief exposure to light for growth. This type of growth has been termed as light activated heterotrophic growth, i.e., LAHG (Anderson and McIntosh, 1991). For LAHG conditions, we illuminated *Synechocystis* cultures daily with 50 μE m^-2^ s^-1^ light for 10 min, which we consider just sufficient to support heterotrophic growth. Under LAHG-xylose conditions, *Synechocystis* strains lacking the xylose catabolic genes did not show any growth (data not shown). This indicates that, the growth exhibited by the strains expressing *xylAB* genes was the result of utilization of xylose and not due to photosynthetic electron transport. Lee et al. (2015) have compared the growth of a *Synechocystis* strain expressing *xylAB* genes with another strain expressing *xylAB* and transporter coding *xylFGH* genes in the presence of four different concentrations of xylose, under modified LAHG conditions wherein the cultures received daily illumination of 50 μE m^-2^ s^-1^ for 1 h. At the end of ninth day under modified LAHG conditions, the latter strain reached higher optical density values only at xylose concentrations of 10 and 50 mM (Lee et al., 2015). We sought to use xylose concentration as low as possible, to evaluate the xylose catabolizing strains on the basis of biomass accumulation and xylose consumption data collected at 6-h intervals for 3 days under mixotrophy and 24-h intervals for 7 days under LAHG conditions. In contrast to the results obtained for the *Synechocystis* strain carrying the XylFGH transporter (Lee et al., 2015), we found that expression of heterologous xylose transporters was able to boost the ability of transformants to utilize xylose, even at low xylose concentrations (5 mM), when it was the sole source of organic carbon. X-Ut2, showed an intermediate ability to utilize xylose, and was more efficient than the X-Ut4 strain. The performance of X-Ut1 and X-Ut3 was the most remarkable (**Figures 7A,B**, **8A–C** and **10A,B**). Thus, among the heterologous transporters studied, GalP was the least efficient. However, we were unable to determine which of the other two transporters, XylE and Glf, was more active, since a bottleneck may exist that influences the catabolic activities of XylAB or enzymes of the PPP of *Synechocystis*. We did not find any significant difference in the biomass yields of X-Ut1 and X-Ut3 strains grown in the presence of 10 and 20 mM xylose under mixotrophic conditions at the end of third day (data not shown). During preliminary experiments performed in our laboratory, we observed that the WT, X-Tr1, X-Tr2, and X-Tr3 strains that lacked the *xylAB* genes and hence the ability to catabolize xylose showed no difference in growth under mixotrophy-xylose (up to 20 mM xylose) and autotrophy conditions (data not shown). These observations indicate the absence of osmotic stress and the presence of an efficient eﬄux system in *Synechocystis*.

Although X-Ut3 was one of the two most efficient strains in terms of dry biomass accumulation and xylose uptake (**Figures 7A,B**, **8A–C** and **10A,B**), it showed the lowest radioactivity among the xylose utilizing strains in the D-[^14^C] xylose uptake study (**Figure 6**). This can be explained by the bidirectional transport ability of Glf (Marger and Saier, 1993). When heterologously expressed in the cyanobacterium *Synechococcus elongatus* PCC 7942, Glf has been shown to export monomeric sugars (Niederholtmeyer et al., 2010). On the other hand, X-Ut1 showed significantly higher levels of radioactivity. These results are not reflected in the actual growth and xylose consumption data (**Figures 6**, **7A,B** and **8A–C**), indicating that a lag phase exists before xylose metabolism is fully established.

When glucose was included in the medium along with xylose, the maximum xylose uptake rates exhibited by the transformants were lower than those when xylose was the sole source of organic carbon (**Figures 10A,B**; Supplementary Table S1). This observation is in accordance with results obtained previously in which a *Synechocystis* strain carrying the *xylAB* genes showed a decrease in xylose consumption in the presence of glucose (Lee et al., 2015). Although glucose was consumed at a much faster rate than xylose (Supplementary Table S1), both sugars were utilized simultaneously with no signs of diauxy (data not shown). These results were expected, since *psbA2* is a light-dependent promoter (Mohamed and Jansson, 1989; Lindberg et al., 2010). Absence of a regulatory region linked to the promoter which can be affected by presence of a sugar and continuous exposure to light caused constant induction of the *psbA2* promoter, hence ensuring the expression of the xylose transporter and catabolic genes even in the presence of glucose. In the presence of 5 mM each of xylose and glucose, the growth and sugar consumption patterns exhibited by the *Synechocystis* strains carrying the *xylAB* genes were found to be similar to each other, which may indicate that the *Synechocystis* strains had reached their maximum capacity to process organic carbon, at least via PPP in the presence of 5 mM glucose. Although diauxy was not observed, the presence of glucose caused a decrease in the maximum xylose uptake rates, with the exception of the X-Ut4 strain under LAHG-mixed sugar conditions (**Figures 10A,B**; Supplementary Table S1). Complete consumption of glucose before that of xylose suggests that glucose was still the preferred sugar of the engineered *Synechocystis* strains.

We believe that there is scope to further improve the ability of the strains to utilize xylose. Codon optimization has been previously shown to improve the expression of heterologous genes in *Synechocystis* (Lindberg et al., 2010; Angermayr et al., 2014; Xue et al., 2014b). Another element that is correlated with the expression level of genes is RBS (Ma et al., 2002). The strength of RBSs can be predicted and improved with the aid of a predictive design tool (Salis et al., 2009). With the help of more powerful RBSs, it will be possible to truly harvest the potential of the *xylAB* and *xylFGH* genes employed in the present and in another recent study (Lee et al., 2015). Approaches such as exploration of genomes of bacteria and algae for potential sugar transporter-catabolic genes, expression of multiple heterologous transporter genes and laboratory evolution could also prove beneficial for obtaining more efficient xylose-utilizing strains.

The strains generated in this work, especially X-Ut1 and X-Ut3, can be used directly or modified further. Introducing the *xylAB* genes into a glycogen synthesis gene *glgC* (*slr1176*) knockout enhanced the production of keto acids, and half of the carbon in the keto acids could be traced to catabolized xylose (Lee et al., 2015). An excess of carbon resulting from the absence of a carbon sink and reduced nutrient availability has been demonstrated to trigger metabolic outflow (Carrieri et al., 2012; Lee et al., 2015). Efficient xylose utilization by engineered *Synechocystis* strains, in addition to the aforementioned strategies, would increase the amount of carbon available for biotechnological conversion. The work reported here represents an important step toward engineering a *Synechocystis* strain that is able to harvest carbon from the second most abundant sugar source in nature and use it to biosynthesize a range of useful compounds.

## Author Contributions

SR and QH conceived the research. SR, YZ, MK, and WM designed and performed the experiments. SR analysed the data. SR and QH prepared the manuscript. All the authors have read and approved of the manuscript.

## Conflict of Interest Statement

The authors declare that the research was conducted in the absence of any commercial or financial relationships that could be construed as a potential conflict of interest.
