# Supplementary material for: Metabolic Engineering and Comparative Performance Studies of Synechocystis sp. PCC 6803 Strains for Effective Utilization of Xylose
Source: Front Microbiol. 2015 Dec 24;6:1484. doi: 10.3389/fmicb.2015.01484 (PMC4689873; doi:10.3389/fmicb.2015.01484)
Supplement: Supplementary file 1 [file Data_Sheet_1.DOCX]

# *Supplementary Material*

**Metabolic engineering and comparative performance studies of *Synechocystis* sp. PCC 6803 strains for effective utilization of xylose.**

Saurabh Ranade^1^, Yan Zhang^2^, Mecit Kaplan^1^, Waqar Majeed^3^, Qingfang He^1*^

^1^ Department of Biology, University of Arkansas at Little Rock, Little Rock, AR, USA

^2^ Biotechnology Research Center, Shandong Academy of Agricultural Sciences, Jinan, Shandong, China

^3^ Center for Integrative Nanotechnology Sciences, University of Arkansas at Little Rock, Little Rock, AR, USA

**^*^ Correspondence:** Qingfang He, University of Arkansas at Little Rock, Department of Biology,

2801 South University Avenue, ETAS 426, Little Rock, AR, 72204, USA.

qfhe@ualr.edu

## 1. Supplementary Figure and Tables

### 1.1 Supplementary Figures

**
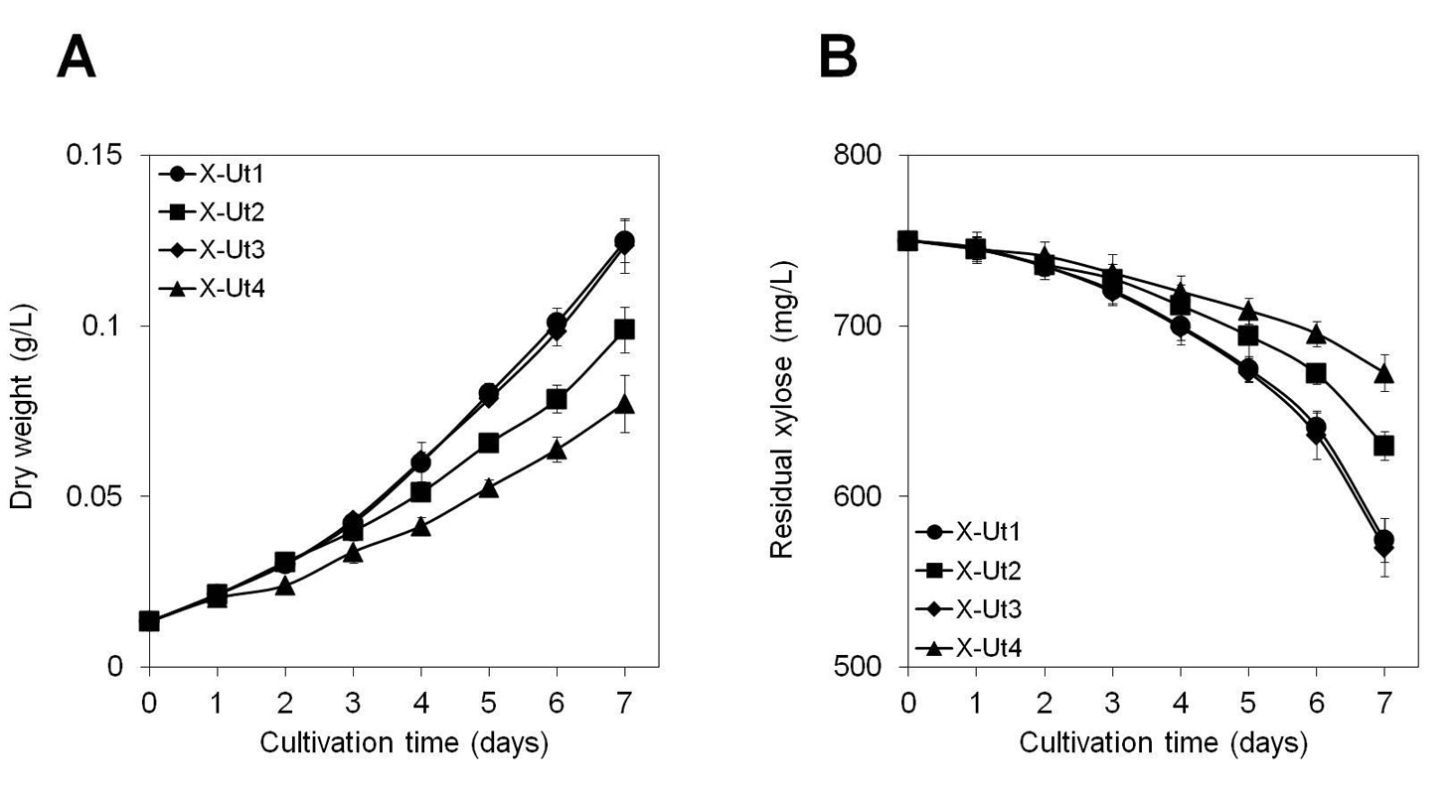
**

#### Supplementary Figure 1. Comprehensive growth and xylose consumption under LAHG conditions in the presence of 5 mM xylose.

*Synechocystis* strains carrying the xylose catabolic genes, *xylAB,* were grown in the presence of 5 mM (750 mg/L) xylose. **(A)** Dry biomass accumulation pattern and **(B)** xylose consumption pattern were studied over a period of 7 days. Measurements were made at 24-h intervals. Data were collected from three biological replicates and presented as means ± standard deviations.


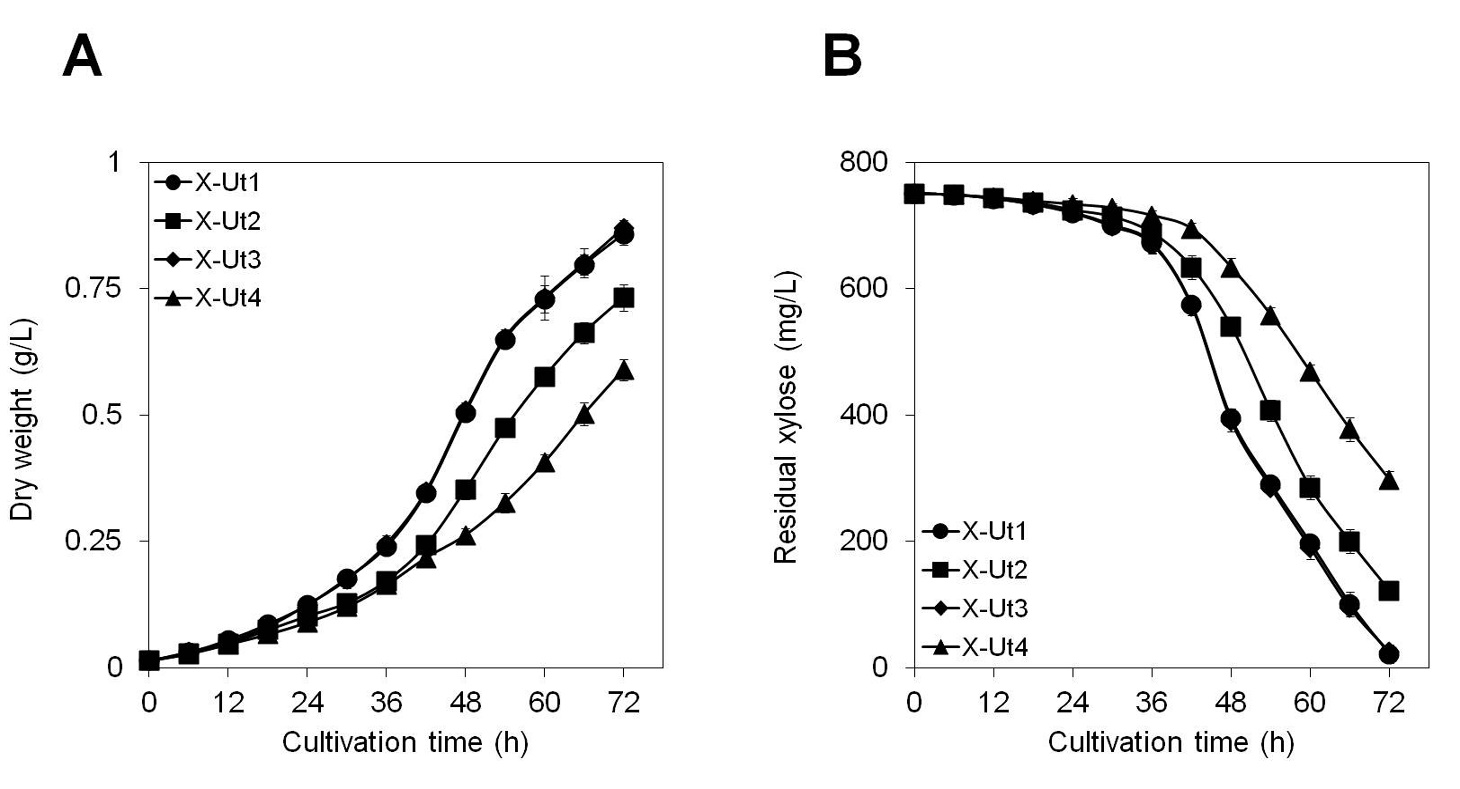


#### **Supplementary Figure 2. Comprehensive growth and xylose consumption under mixotrophy in the presence of 5 mM xylose**.

*Synechocystis* strains carrying the xylose catabolic genes, *xylAB,* were grown in the presence of 5 mM (750 mg/L) xylose. **(A)** Dry biomass accumulation pattern and **(B)** xylose consumption pattern were studied over a period of 3 days. Measurements were made at 6-h intervals. Data were collected from three biological replicates and presented as means ± standard deviations.

### 1.2 Supplementary Table

#### Supplementary Table 1. Maximum sugar uptake rates in the presence of 5 mM each of xylose and glucose.

| Strain | Maximum sugar uptake rate (mg sugar g^-1^ DW h^-1^) | | | |
| --- | --- | --- | --- | --- |
|  | LAHG | | Mixotrophy | |
|  | Xylose | Glucose | Xylose | Glucose |
| X-Ut1 | 17.24 ± 1.24 | 71.32 ± 5.03 | 25.82 ± 1.28 | 113.42 ± 7.85 |
| X-Ut2 | 16.59 ± 1.00 | 72.06 ± 3.35 | 26.14 ± 1.39 | 111.67 ± 4.86 |
| X-Ut3 | 17.00 ± 1.15 | 71.54 ± 2.25 | 26.05 ± 1.04 | 111.51 ± 5.20 |
| X-Ut4 | 15.47 ± 1.33 | 70.32 ± 3.15 | 26.22 ± 2.29 | 114.15 ± 9.42 |

*Synechocystis* strains carrying the xylose catabolic genes, *xylAB,* were grown in the presence of 5 mM (750 mg/L) xylose and 5 mM (900.8 mg/L) glucose. Maximum xylose and glucose uptake rates were calculated as mg sugar g^-1^ dry weight (DW) h^-1^ using a previously described formula (Munyon and Merchant, 1959). For all the strains, maximum xylose uptake rates were observed between the 6^th^ and 7^th^ day under LAHG conditions and between the 42^nd^ and 48^th^ hour under mixotrophy. Similarly, for all the strains, maximum glucose uptake rates were observed between the 3^rd^ and 4^th^ day under LAHG conditions and between the 30^th^ and 36^th^ hour under mixotrophy. Raw data were collected from three biological replicates. Uptake rate values obtained are presented as means ± standard deviations.

## 2. References

Munyon, W. H., and Merchant, D. J. (1959). The relation between glucose utilization, lactic acid production and utilization and the growth cycle of L strain fibroblasts. *Exp. Cell Res*. 17, 490-498.doi:10.1016/0014-4827(59)90069-2.
